# UV-C-Activated Riboflavin Crosslinked Gelatin Film with Bioactive Nanoemulsion for Enhanced Preservation of Fresh Beef in Modified Atmosphere Packaging

**DOI:** 10.3390/foods13213504

**Published:** 2024-10-31

**Authors:** Jumana Mahmud, Peter Muranyi, Stephane Salmieri, Shiv Shankar, Monique Lacroix

**Affiliations:** 1 INRS Armand-Frappier Health Biotechnology Research Centre, Research Laboratories in Sciences, Applied to Food (RESALA), Canadian Irradiation Centre (CIC), MAPAQ Research Chair in Food Safety and Quality, Institute of Nutrition and Functional Foods (INAF), 531 Des Prairies Blvd, Laval, QC H7V 1B7, Canada; jumana.mahmud@inrs.ca (J.M.); stephane.salmieri@inrs.ca (S.S.); shiv.shankar@inrs.ca (S.S.); 2 Fraunhofer Institute for Process Engineering and Packaging IVV, Giggenhauser, Str. 35, 85354 Freising, Germany; peter.muranyi@ivv.fraunhofer.de

**Keywords:** UV-C irradiation, riboflavin, photo-crosslinking, nanoemulsion, active film, modified atmosphere packaging, beef

## Abstract

This study explores a new eco-friendly approach for developing bioactive gelatin films using UV-C irradiation-induced photo-crosslinking. Riboflavin, a food-grade photoinitiator, was selected at an optimal concentration of 1.25% (*w*/*w*) for crosslinking gelatin under UV-C exposure for 4 to 22 min. Physicochemical analyses revealed enhanced tensile strength, reduced water vapor permeability, and lower water solubility in films crosslinked for up to 13 min. FTIR analysis demonstrated significant molecular changes, confirming the formation of crosslinking connections in gelatin–riboflavin films. Antimicrobial nanoemulsion (NE) (0.5, 0.75, 1% *v*/*v*) was incorporated into crosslinked films and applied to fresh beef. The 1% NE film exhibited the strongest antimicrobial effect, extending shelf-life by 20 days. In vitro release study confirmed Fickian diffusion behavior in the 1% NE film. This study also investigated the synergy between 1% NE film and three different types of modified atmosphere packaging (MAP) on the microbiological and physicochemical properties of beef for 26 days. The best results were achieved with 1% NE film under MAP1 and MAP2, which preserved meat redness and prevented lipid oxidation, extending the shelf-life up to 26 days. Therefore, UV-C irradiation-induced crosslinked bioactive film combined with high-oxygen MAP offers a promising solution for prolonging the shelf-life of beef.

## 1. Introduction

Beef is a widely consumed food item, ranking third globally in per capita meat consumption. A report by Grand View Research Inc. (San Francisco, CA, USA, 2019) estimates that the global beef market will grow at a compound annual rate of 3.1%, reaching a value of USD 383.5 billion by 2025 [1]. Nevertheless, beef is rich in nutrients creating favorable conditions for the proliferation of various spoilage bacteria such as *Pseudomonas* spp., lactic acid bacteria (LAB), and *Brochothrix thermosphacta* [2]. Due to its high perishability, extending the shelf-life of beef presents a significant obstacle for the meat industry. During storage and distribution, beef quality declines as a result of microbial activity, discoloration, and the oxidation of lipids and proteins [3]. To combat these issues, the industry uses high-oxygen modified atmosphere packaging (high-O_2_ MAP) with 80% O_2_ + 20% CO_2_, which maintains an appealing red color and extends the shelf-life to about 10 days at refrigerated temperatures, providing a consistent and cost-effective product for retail [4]. Carbon dioxide (CO_2_) is utilized to inhibit bacterial growth, while oxygen helps maintain the color of the product [5]. However, high-O_2_ MAP can increase the oxidation of meat and lead to discoloration, off-flavors, premature browning, and reduced tenderness that shortens shelf-life [6]. Optimizing the appropriate gas mixture is crucial for ensuring food safety.

Considering the limitations of high-O_2_ MAP, antimicrobial films have introduced an innovative approach to the concept of active packaging [7]. Notably, active packaging that releases antimicrobial volatile compounds, such as essential oils (EOs) and plant extracts, has attracted significant scientific attention, as these substances can modify the package atmosphere during storage and distribution [8,9]. Active packaging combined with MAP is seen as a potential method for preserving food due to two main factors: alterations in packaging atmosphere can influence microbial growth differently, and the inclusion of bioactive compounds with antioxidant properties can mitigate oxidation, thereby prolonging shelf-life [10].

EOs and plant extracts containing phenolic compounds and flavonoids meet the rising demand for natural additives with antimicrobial properties [11,12]. EOs act by disrupting membranes, causing leakage of intracellular components, and inflicting metabolic damage, ultimately resulting in cell death. To optimize effectiveness and minimize concerns, careful selection and mixture design are recommended [13]. However, EOs face limitations due to volatility, solubility, and degradation, which can be addressed through encapsulation in delivery systems like nanoemulsion (NE). NE generally exhibits remarkable stability, resisting both gravity-based partitioning and the aggregation of droplets [14]. Their nanoscale size and enhanced diffusion properties enable the controlled release of antimicrobial agents while minimizing harmful effects on the sensory attributes of food products [15].

Different types of biopolymers offer a sustainable alternative to traditional petroleum-based packaging materials used in active packaging systems due to their biodegradability and renewability, significantly reducing environmental impact [16]. The United Nations Sustainable Development Goals (SDGs) for 2030 offer a roadmap for global development, focusing on ecological, economic, and social sustainability [17]. Three key SDGs—SDG 9 (Industry, Innovation, and Infrastructure), SDG 12 (Responsible Consumption and Production), and SDG 13 (Climate Action)—align with research aimed at developing sustainable packaging systems. SDG 9 promotes innovation in the meat industry through advanced bioactive films for food packaging. SDG 12 focuses on ensuring sustainable consumption and production patterns, which is highly relevant to food packaging. Bioactive films contribute to sustainable production by reducing reliance on non-renewable resources like plastics and minimizing packaging waste. Moreover, by extending the shelf-life of meat products, these films can help reduce food waste, which is a major issue in the food supply chain. SDG 13 (Climate Action) highlights the urgency of addressing climate change [18]. Gelatin (GT), a protein from partially hydrolyzed collagen derived from animal wastes/by-products, is valued in the food industry for its low cost, biocompatibility, film-forming ability, low water vapor transmission, oxygen barrier properties, and effectiveness in carrying bioactive components [19]. However, its application as a packaging film is limited by its weak mechanical properties and high solubility in water [20]. To address these issues, methods such as adding nanoscale particles and fibers and using crosslinking techniques (enzymatic, chemical, or UV irradiation) have been explored [21,22].

Enzyme and chemical crosslinking in food packaging can be costly and potentially toxic [23]. Alternatively, low-energy UV irradiation-induced crosslinking with riboflavin presents an eco-friendly, non-toxic solution, contributing to the growing demand for sustainable active food packaging [24,25]. Riboflavin (RF) is water-soluble vitamin B2, one of the natural food-grade photoinitiators utilized in this process [25]. Studies have demonstrated that a synergistic effect of irradiation and RF can produce radicals, leading to crosslinking and intermolecular connections in proteins [26,27,28]. When RF is exposed to UV light, it forms reactive singlet oxygen (^1^O_2_) and transitions to highly reactive triplet-excited oxygen (^3^O_2_), which modifies biomolecules and amino acid side chains within proteins [29]. This process enables RF to engage in type I and II photosensitized oxidation reactions, facilitating crosslinking in the GT matrices [30]. In type I reactions, RF-free radicals combine with ^1^O_2_, leading to oxidation products. In type II reactions, RF transfers energy to oxygen molecules to produce ^1^O_2_, which then interacts with the substrate, resulting in oxidation products. For example, it proceeded via rapid electron transfer or proton-coupled electron transfer primarily from the side chains of the amino acids tyrosine, histidine, tryptophan, and cysteine in proteins [31]. Photosensitized reactions generally involve a combination of various reactive oxygen species (ROS) such as ^1^O₂, hydroxyl radicals (·OH) and superoxide anions (O_2_^−^), as well as cationic and anionic radicals of RF [28]. Additionally, UV rays can affect the structure of GT, opening amino acid functional groups for crosslinking with RF, resulting in stronger interactions between GT and RF [25].

Based on the above considerations, this study aimed to explore how the shelf-life of beef could be extended by using crosslinked bioactive film alone or in combination with MAP at varying O_2_/CO_2_ ratios, by monitoring microbial growth, color, and lipid oxidation.

## 2. Materials and Methods

### 2.1. Materials

GT from porcine skin (~175 bloom, Type A), RF, Tween^®^ 80, and Span^®^ 80 were obtained from Sigma-Aldrich Canada Co., (Oakville, ON, Canada). Glycerol was bought from Fisher Scientific (Ottawa, ON, Canada). Mediterranean EO, German thyme EO, and Vietnamese Cinnamon EO were purchased from Bio Lonreco (Montreal, QC, Canada) and citrus extract was supplied by Zayat Aroma Inc. (Bromont, QC, Canada).

### 2.2. Preparation of Nanoemulsion (NE)

The NE was prepared using the antimicrobial formulation (Supplementary File) described by Mahmud et al. [13]. This involved blending Mediterranean EO, German thyme EO, and Vietnamese cinnamon EO with citrus extract, utilizing the emulsifiers Tween^®^ 80 and Span^®^ 80. The mixture was adjusted to an HLB value of 12 and a surfactant-to-oil ratio of 0.75:1 and mixed by Ultra-Turrax (15,000 rpm for 1 min) at ambient temperature. The resulting coarse emulsion was subjected to high-pressure homogenization using a microfluidizer at 15,000 psi for 4 cycles. The selection of this NE was based on earlier work conducted by Mahmud et al. [32].

### 2.3. Preparation of the Crosslinked Active Films

Films were produced through a casting method, where 4% (*w*/*v*) GT was dispersed in distilled water under continuous stirring for 30 min at 45 °C. Different amounts of RF (0.3, 0.5, 0.75, 1.25% *w*/*w* of GT) were added into the GT solution and stirred at 45 °C for 15 min. Glycerol (30% *w*/*w* of GT) was added as a plasticizer and stirred for another 15 min at 45 °C to achieve a homogenous solution. Lastly, different concentrations of NE (0.5, 0.75, 1% *v*/*v*) were added to the GT-RF dispersion under magnetic stirring. The polymeric dispersion was purged with nitrous oxide (N_2_O) for 20 min prior to irradiation. Then, 20 mL of the polymeric dispersion poured into glass Petri dishes and exposed to UV-C light (280–100 nm) for a period of 4 to 22 min using a BS-04 UV irradiator (Opsytec Dr. Gröbel GmbH, Ettlingen, Germany) equipped with 20 UV-C lamps (18 W each; UV output: 4.5 W) and a UV-Mat dosimeter for a time/dose-controlled, uniform UV-C irradiation process. The chamber geometry (footprint) was 60 × 60 × 28 cm (L × D × H) and the irradiance was equal to 8 mW/cm^2^. The polymeric dispersion was dried at 23 ± 2 °C for 24 h and then conditioned in a desiccator before the analyses of their physico-mechanical properties.

### 2.4. Characterization of Films

Mechanical properties, including tensile strength (TS, MPa), tensile modulus (TM, MPa), and elongation at break (Eb, %), were assessed by following the ASTM D638-99 test method [33]. Measurements were conducted using a Universal Testing Machine (UTM) H5KT from Tinius Olsen Testing Machine Co., Inc. (Horsham, PA, USA) and Test Navigator program (ver. 7.02.11). Films were measured in tensile mode with an initial grip separation of 30 mm, a crosshead speed of 50 mm/min, and a load cell of 100 N. Ten measurements were performed for all films, and the mean values are reported.

Water vapor permeability (WVP) of films was performed according to the ASTM E96/E96M-16 procedure [34]. Films were securely sealed onto EZ^®^ Vapometer permeability cups (model 68–3000; Twhing-Albert Instrument Co., West Berlin, NJ, USA) containing anhydrous CaCl_2_ (30 g). WVP values were then calculated using a combined model based on Fick and Henry’s laws of gas diffusion through coatings and films, as described by Equation (1):

WVP (g·mm/m^2^·day·kPa) = Δw·x/A·ΔP (1)

where ∆w is the weight gain of the cell (g) after 24 h, x is the film thickness (mm), A is the area of exposed film (31.67 × 10^−4^ m^2^), and ∆P is the differential vapor pressure of water through the film (∆P = 3.2823 kPa at 25 °C).

The water solubility (WS) of the films was evaluated using the procedure described in Taghizadeh et al. [25]. Film samples (2 × 2 cm) were cut, weighed, and then immersed in distilled water (50 mL). The samples were stirred for 24 h at room temperature. After the soaking period, the film pieces were removed from the water, dried at 105 °C for 1 h, and then re-weighed. The WS of the films was determined using Equation (2):

WS% = (Wi − Wf)/Wi × 100(2)

where Wi is the initial weight and Wf is the weight of the undissolved film after drying.

Fourier transform infrared (FTIR) analysis was conducted according to the procedure outlined in Huq et al. [35], using a Spectrum One spectrophotometer (PerkinElmer, Woodbridge, ON, Canada) equipped with an attenuated total reflectance (ATR) device for solids analysis and a high-linearity lithium tantalate (HLLT) detector. Analyses were performed within 4000–650 cm^−1^ with 64 scans recorded at a 4 cm^−1^ resolution. After attenuation of total reflectance and baseline correction, spectra were normalized with a limit ordinate of 1.5 absorbance units.

### 2.5. Apparent Release Kinetics (In Vitro)

The release kinetics of bioactive compounds from the crosslinked GT-RF films (13 min UV-C treated) were determined following the method of Ben-Fadhel et al. [36] with some modifications. Firstly, 500 mg film samples were dissolved in 100 mL of 95% ethanol as a simulant for fatty foodstuffs [37] and then stored at 4 °C. At each sampling time, 1 mL of film solution was removed, and an equivalent amount of the corresponding food simulant was added back to ensure a consistent volume. The absorbance of each sample was read at 232 nm in a UV-Vis Spectrophotometer Scinco S-3100 (Betatek Inc., North York, ON, Canada), and the concentrations of bioactive compounds contained in NE were calculated using a standard curve.

The cumulative release was calculated by the following Equation (3):

Cumulative percentage release (%) = (Volume of sample withdraw (mL))/(Bath volume) × *P* (*t* − 1) + *Pt* (3)

where:

*Pt*: Percentage release (*w*/*v*) at time t;

*P*(*t* − 1): Percentage release (*w*/*v*) at a time before ‘*t*’.

To explain the kinetics and mechanisms of bioactive compound release from the crosslinked film, the in vitro release data were analyzed using the Korsmeyer–Peppas model as outlined in Equation (4).

*Mt*/*M∞* = *k_kp_ t^n^* (4)

where *Mt*, *M∞*, *k_kp_*, *n*, and *t* represent the total bioactive compounds released at time *t*, the quantity of bioactive compounds released at infinite time, the release constant, the release exponent, and sampling time, respectively. The ‘*n*’ exponent indicates different release mechanisms of the bioactive compounds from a polymeric matrix. When *n* ≤ 0.45, the release corresponds to Fickian diffusion. For values between 0.45 and 0.89 (i.e., 0.45 < *n* < 0.89), bioactive compound release occurs through anomalous transport (drug diffusion in the hydrated matrix and polymer relaxation). At *n* = 0.89, the process indicates zero-order release, mostly driven by a swelling phenomenon. In cases where *n* > 0.89, the transport is classified as non-Fickian, typically involving erosion or polymer relaxation [34,35]. The kinetic rate constant (*k*) was determined using the linear regression of the log of the release percentage ((*Mt/M∞*) × 100) against log time (h), ensuring that the correlation coefficient (*R*^2^) was greater than 0.9.

### 2.6. In Vitro Antimicrobial Test

The inhibitory capacity (IC, %) of the bioactive films was determined by a micro-atmosphere agar diffusion assay based on a procedure of Hossain et al. [38]. Each film (3–4 cm^2^) was placed in the lid of a Petri dish (center position) containing different agar media depending on the type of bacteria: de Man Rogosa Sharpe agar for *Lactobacillus sakei*, *L. curvatus*, and *Leuconostoc mesenteroides*; Brain-Heath Infusion agar for *Carnobacterium divergens*; streptomycin thallous acetate actidione agar for *B. thermosphacta*; and cetrimide agar for *Pseudomonas aeruginosa* (Alpha Biosciences, Baltimore, MD, USA). Then, 100 µL of each activated bacterial culture (10^6^ CFU/mL) was spread onto the surface of agar plates. Petri dishes (92 × 16 mm; Sarstedt Inc., Saint-Leonard, QC, Canada) were covered with Parafilm^®^ to inhibit evaporation of volatile EOs and then incubated at 30 °C for 24 h. The inhibition zone was measured using a Traceable^®^ Carbon Digital caliper (Fisher Scientific Ltd.).

The IC (%) was calculated according to Equation (5):

IC (%) = [Diameter inhibition zone (mm)/Diameter of Petri dish (mm)] × 100(5)

The diameter of Petri dishes used was standardized at 83 mm.

### 2.7. In Situ Antimicrobial Effect of Bioactive Films—Shelf-Life of Meat

#### 2.7.1. Antimicrobial Effect of Bioactive Films in Air Packaging

Fresh sliced beef was supplied by Montpak International (Laval, QC, Canada), divided into 4 groups. Next, the beef samples covered with films were placed on polypropylene trays, sealed under air (78.1% N_2_, 20.9% O_2_, and 0.036% CO_2_) and stored at 4 °C. Sample groups were prepared as follows: (1) control: film without NE (C-air); (2) film containing 0.5% NE (0.5% NE-air); (3) film containing 0.75% NE (0.75% NE-air); and (4) film containing 1% NE (1% NE-air). Microbiological analyses of all beef samples were conducted at specific intervals until the bacterial count reached the acceptable limit of 7 log₁₀ CFU/g [39].

#### 2.7.2. Antimicrobial Effect of Bioactive Films in Combination with MAP

Beef samples with active films were placed on polypropylene trays and then sealed under varying MAP conditions which consisted of 80% O_2_ + 20% CO_2_ (MAP1), 70% O_2_ + 30% CO_2_ (MAP2), and 60% O_2_ + 20% CO_2_ + 20% N_2_ (MAP3). All tray-packaged samples were divided into 8 groups as follows: (1) control: film without NE (C-air); (2) active film under air (1% NE-air); (3) film without NE + MAP1 (MAP1); (4) film without NE + MAP2 (MAP2); (5) film without NE + MAP3 (MAP3); (6) active film + MAP1 (1% NE-MAP1); (7) active film + MAP2 (1% NE-MAP2); and (8) active film + MAP3 (1% NE-MAP3). Microbiological analyses of all beef samples were conducted at specific intervals until the bacterial count reached the acceptable limit of 7 log₁₀ CFU/g [39].

#### 2.7.3. Microbiological Analysis

A 10 g beef sample was mixed with 90 mL of sterile peptone water (0.1%) and homogenized for 2 min using a Lab-blender 400 stomacher (Seward Medical, London, UK). Serial dilutions were prepared and spread evenly onto sterile Petri dishes filled with Tryptic soy agar, which were then placed in an incubator for 24 h at 35 ± 1 °C to determine the total viable count (TVC). Total LAB, *B. thermosphacta*, and *Pseudomonas* spp. were determined using specific agar media previously mentioned in Section 2.6 and then incubated at 25 °C for 48 h. After incubation, the colony-forming units (CFU) were counted using a magnifier [40].

### 2.8. Determination of Thiobarbituric Acid Reactive Substances (TBARSs)

Lipid oxidation in meat was determined using the method outlined by Oussalah et al. [41]. The results are reported as mg malondialdehyde (MDA)/kg.

### 2.9. Color Parameters of Meat

The color values L* (lightness), a* (redness), and b* (yellowness) were checked immediately after opening the beef package by using a Color Reader Konica Minolta CR-10 Plus (Folio Instruments Inc., Kitchener, ON, Canada), following a procedure by Masoomian et al. [42].

### 2.10. Statistical Analysis

All experiments were conducted in triplicate (*n* = 3). The data underwent analysis of variance, and mean differences were assessed with Duncan’s test. Differences between means were significant when *p* ≤ 0.05. IBM SPSS Statistics 26 (IBM Corp., Armonk, NY, USA) was utilized for all data analyses.

## 3. Results and Discussion

### 3.1. Mechanical Properties

TS, TM, and Eb are key mechanical properties for assessing polymer films in food packaging, as they reflect the material’s capacity to withstand stress while preserving structural integrity throughout processing, transportation, and storage [43]. RF at 1.25% (*w*/*w*) improved the mechanical properties of GT films, showing better TS (28.05 MPa), TM (294.49 MPa), and Eb (80.55%) compared to pure GT films (without RF) (Table 1). This improvement may have been facilitated by the interaction between GT and RF, which can create a dense network structure due to their good distribution within the GT-RF film. A previous study by Su et al. [44] confirmed the even dispersion of RF throughout the chitosan film using scanning electron microscopy (SEM) and found that the addition of RF improved various film characteristics, including thickness, mechanical properties, solubility, and water barrier properties. Therefore, 1.25% (*w*/*w*) was chosen as the optimal RF concentration for crosslinking under UV-C irradiation. The physicochemical properties of pure GT and crosslinked GT-based films under different UV-C treatments are presented in Table 2.

TM for pure GT films (uncrosslinked without UV-C) was 125.9 MPa, while the addition of RF combined with UV-C irradiation at different irradiation times significantly increased TM to 323.5 MPa (4 min), 427.6 MPa (9 min), and 573.1 MPa (13 min) (*p* ≤ 0.05) (Table 2). Therefore, the film RF-13min UV-C showed the highest TM, which was significantly increased (*p* ≤ 0.05) by 94% compared to the RF-crosslinked film without UV-C treatment (294.5 to 573.1 MPa). These results indicate that RF in combination with UV-C irradiation adequately increased the film stiffness through a crosslinking mechanism. A significant increase (*p* ≤ 0.05) in TS was also observed after 4 to 13 min of exposure to UV-C irradiation, which was about 21–66% higher than that of the crosslinked film without exposure to UV-C treatment (46.7 MPa for RF-13min UV-C compared to 28.1 MPa for RF without UV-C). Photo-crosslinking could restrict the mobility of polymer molecules, and thus TS and TM showed an increasing trend with an extended UV-C exposure time [45]. Taghizadeh et al. [22] reported that TS of a UV-treated GT-RF film was about 8 times greater than that of the film without RF and irradiation treatment.

However, a significant decrease in TS was observed after 17 min of irradiation, possibly attributed to polymer degradation induced by UV-C irradiation [45]. According to Zain et al. [46], the correlation between increased irradiation time and improved TS is not always consistent, potentially because of the induction of damage to the polymer chains during the irradiation treatment. Additionally, introducing higher amounts of photo-additives (>6% of polymer dry basis) can impede the penetration of UV light and diminish the crosslinking reaction, leading to a decline in TS. Following that, incorporating NE into crosslinked films slightly decreased TS from 46.7 MPa (RF-13min UV-C) to 43.3, 40.7, and 38.7 MPa for 0.5%, 0.75%, and 1% NE, respectively. Despite this, TS values were still 1.8 to 2 times higher than those of pure GT films. Similarly, TM dropped from 573.1 MPa (RF-13min UV-C) to 470, 410, and 333 MPa, but these values were 1.6 to 2.7 times higher than those of pure GT films. Despite some reduction, NE-incorporated crosslinked films maintained greater TS and TM compared to pure GT films. Based on our results obtained for TS and TM, RF-13min UV-C film can be assigned to an optimal crosslinking process.

The addition of RF significantly decreased (*p* ≤ 0.05) Eb of the films without NE by 18.6% (86.01% for pure GT film to 67.4% for RF-13min UV-C film). The reduction in Eb is primarily attributed to the increased stiffness of films due to the photochemical crosslinking instead of the particle reinforcement of RF [28]. Inversely, the incorporation of 1% NE in films significantly increased Eb up to 98.0% (*p* ≤ 0.05) compared to films without NE (*p* ≤ 0.05), due to the plasticizing effect of EOs and citrus extract, which enhanced film elasticity (a decrease in rigidity equivalent to an increase in flexibility). It was reported that phenolic components containing hydroxyl and carbonyl groups found in EOs and citrus extract interact hydrophobically with the NH_3_ groups of GT, leading to such an elongation improvement in biomaterials [47]. The findings from the above results allow us to deduce that the introduction of RF into GT-based films under UV-C irradiation led to considerable enhancements in TS and TM due to GT-RF polymer crosslinks, whereas the addition of NE improved the ductility of films.

### 3.2. Water Vapor Permeability (WVP)

The low WVP of the films is of high relevance for the longevity of a food product as it serves to minimize the exchange of moisture between the interior and exterior packaging conditions [48]. The WVP values of pure GT film (control) and crosslinked films are presented in Table 2. Generally, pure GT films showed a high WVP due to their non-linear structure and highly hydrophilic groups [49]. Increasing the UV-C irradiation times significantly decreased (*p* ≤ 0.05) the WVP of films. An increase in the water barrier was noted in films treated with RF-13 min of UV-C exposure compared to other treatments, with a minimum value of 6.7 g·mm/m^2^·day·kPa. This value corresponds to a 67% decrease compared to pure GT films and a 60% decrease compared to films containing RF without UV-C. Thus, crosslinked GT restricts hydrogen bond formation and water absorption within the biopolymer, thereby reducing water entrance within the film into the food matrix. Additionally, the compact structure of crosslinked GT decreases the rate at which water vapor is absorbed and migrates within the film, leading to a lower WVP [47]. According to Bai et al. [50], hyaluronan/polyvinyl alcohol films with styryl pyridinium groups were able to create a network by photo-dimerization. This network created a tortuous path that obstructed the passage of water molecules. Shahbazi et al. [51] also assessed photo-crosslinked carboxymethyl cellulose films and found a lower WVP compared to films crosslinked through chemical methods. Based on such correlated results obtained for TS, TM, and WVP, RF-13min UV-C film can be assigned to an optimal crosslinking process.

Conversely, the reason for an increasing WVP at 17 and 22 min of UV-C irradiation might be the scission of GT chains, which expanded the spaces between the chains and consequently led to higher WVP. Furthermore, the addition of 1% NE significantly (*p* ≤ 0.05) decreased WVP of films to 3.7 g·mm/m^2^·day·kPa, which was about 81% lower than that of pure GT film. This reduction is mainly due to the hydrophobic nature of EOs and flavonoids in citrus extract, which alters the hydrophilic/hydrophobic balance of films even at a low ratio, limiting water vapor transfer by increasing mass transfer tortuosity. Similar effects were observed in hake protein-based films incorporating thyme EO, where increasing EO levels decreased WVP [52].

### 3.3. Water Solubility (WS)

Film solubility is one of the most significant indicators of resistance to water [53]. GT-based films, due to their hydrophilic characteristics, exhibit high WS, which poses a limitation on their suitability for packaging purposes. Our study showed that UV-C exposure durations (4 to 22 min) significantly decreased the solubility of GT films (Table 2). Film formulation treated in the presence of RF-13min under UV-C irradiation showed a significant reduction (*p* ≤ 0.05) in WS (11.9%) compared to the pure GT film (100%) and the other crosslinked films (13.1–25.3%). This decrease in WS obtained by UV-C crosslinking of films can be explained by the formation of a highly semi-interpenetrated network and a decrease in hydrophilic amino acids, which prevented water penetration into the films [28]. The results obtained in this study are in agreement with Bhat and Karim [54], who showed that UV-C exposure led to the formation of covalent bonds between GT and ribose, subsequently reducing the water solubility of GT-based films. These results are also consistent with those obtained in this study for WVP measurements.

Moreover, the RF-1%NE-13min UV-C film achieved the lowest WS value (5.5%). This additional reduction can be attributed to hydrogen bonding between protein NH_3_ groups and phenolic compounds in EOs and citrus extract, forming a denser film with decreased water affinity [55]. Lowering WS is crucial as it minimizes the release of active compounds from films, thereby potentially extending the shelf-life of food products [56]. As a corroborating observation of film characterization, the film formulation RF-13min UV-C with different NE contents was selected for subsequent microbiological tests.

### 3.4. FTIR Analysis

**Figure 1:**
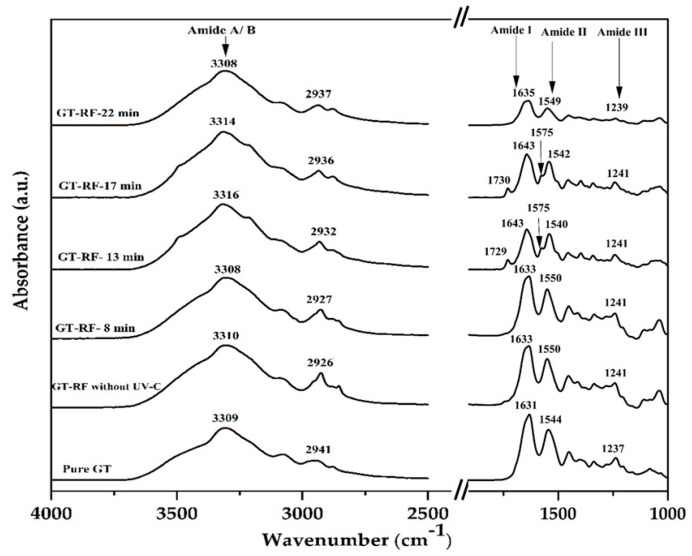
FTIR spectrum of UV-C irradiated crosslinked GT-RF films.

Figure 1 represents the FTIR spectra of pure GT film and GT films crosslinked by RF/UV-C irradiation at different exposure times. Changes in vibration bands show potential molecular interactions that might be associated with RF-mediated crosslinking of GT when exposed to UV-C irradiation. The FTIR spectrum of pure GT film displayed some characteristic absorption bands: 3600–3100 cm^−1^ (–OH stretching vibration from Amide A and –NH/NH_3_+ stretch from Amide B), 2941 cm^−1^ (C–H antisym and sym stretch), 1631 cm^−1^ (C=O stretch coupled to C–N stretch and N–H bending from Amide I band), 1544 cm^−1^ (C–N stretch coupled to N–H bending from Amide II band), and 1237 cm^−1^ (N–H bending in-plane coupled to C–N stretch from Amide III band) as previously reported [57,58]. It was observed that the peak intensity at wavenumbers from 3500 to 2700 cm^−1^ was higher for crosslinked films compared to the pure GT film (control) and GT-RF film without UV-C irradiation. Likewise, UV-C irradiation caused an obvious increase in the -OH/-NH stretching band accompanied by a shift to higher frequencies from 3309 to 3316 cm^−1^, suggesting a crosslinking effect on the protein primary structure and conformational changes.

Crosslinked films during 13 and 17 min of UV-C exposure led to an observable shift of absorption towards higher wavenumbers from 1633 to 1643 cm^−1^ of the Amide Ⅰ band as compared to the GT-RF film without irradiation. Furthermore, a slight shift of the Amide III band, from 1237 to 1241 cm^−1^, was also observed, indicating changes in GT structure. Similarly, Wang et al. [28] and Taghizadeh et al. [25] reported a shift to higher wavenumbers of the Amide A, I, and III bands of GT protein due to UV/RF-mediated crosslinks.

In addition, in the fingerprint infrared region, two new covalent link vibrations appeared in the spectra of crosslinked films exposed to UV-C for 13 and 17 min. Indeed, a new band appeared at 1729 cm^−1^ that is related to C=O stretching due to the presence of the imidazolone group (i.e., carbonylated imidazole group) in the structure of RF. A second new band was observed at 1575 cm^−1^, which might correspond to N-H bending and aromatic ring stretching of imidazolone groups, based on the mechanism of reaction, implying imidazole groups of histidine and tryptophan moieties from the protein reported previously by McCall et al. [59]. RF can prompt crosslinking of protein molecules when exposed to UV-C irradiation due to the participation of histidine, hydroxyproline, hydroxylysine, tyrosine, and threonine in crosslinking reactions. This histidine can facilitate the conversion of imidazole moieties into an electrophilic imidazolone through the activation of ROS and interacts with nucleophilic amino acids to form covalent crosslinks [29,60].

**Figure 2:**
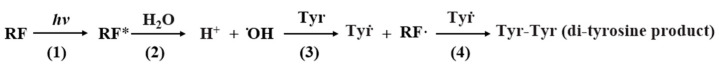
Riboflavin-induced crosslinking of proteins through di-tyrosine formation.

Additionally, new covalent bonds could be created where photodynamic alteration of tyrosine likely aids in the RF-induced crosslinking of proteins through di-tyrosine formation (Figure 2). Typically, ionizing radiation of aqueous protein solutions in the presence of N_2_O generates hydroxyl radicals (·OH) through the process of water radiolysis. Under UV-C irradiation, RF absorbs UV-C photons and becomes excited (RF*) (Figure 2, step 1). The excited RF* can then interact with water molecules to produce ROS such as ·OH (Figure 2, step 2). Sulfur-containing and aromatic amino acids are more susceptible to reactions with ROS compared to aliphatic amino acids. For instance, tyrosine is particularly vulnerable to ·OH attack. ·OH can oxidize tyrosine, leading to the formation of a tyrosyl radical (Tyr·) and an RF anion radical (RF) (Figure 2, step 3). These tyrosyl radicals may subsequently react with other tyrosyl radicals or tyrosine molecules, resulting in the production of di-tyrosine (Tyr-Tyr) products (Figure 2, step 4) [60]. These compounds contain covalent bonds linking their phenolic components. Within these products, 2′,2-biphenol di-tyrosine is notably significant. Di-tyrosine formation is more probable between two distinct protein chains (intermolecular crosslinking) instead of occurring within the same protein chain (intramolecular crosslinking). The intermolecular formation of di-tyrosine is one mechanism for protein aggregation, although other crosslinks can also form [61]. The efficiency of polymerization induced by irradiation is influenced by various factors, including dose rate, the presence of antioxidants, oxygen levels, and the molecular structure of biopolymers. Oxygen and free radicals from irradiation can degrade proteins by forming peroxyl radicals [28].

Thus, in this study, N_2_O was purged in the film-forming solution before irradiation. Moreover, prolonged irradiation can alter the distribution of protein molecular sizes, resulting in the formation of high-molecular-weight particles. This occurs through initial protein fragmentation followed by subsequent aggregation [62]. According to Cho et al. [63], UV irradiation resulted in alterations to the molecular size of β-lactoglobulin, increasing from 18.4 kDa to 35 kDa as the irradiation time increased, specifically after 16 and 32 h of irradiation. Similarly, gamma irradiation at 32 kGy enhanced protein aggregation due to the formation of di-tyrosine bonds, resulting in a more than 15-fold increase in molecular weight [64].

FTIR results corroborate those of physico-mechanical analyses and confirm RF-sensitized protein crosslinking and structural changes in the GT polymer network under UV-C irradiation, with the optimal condition reached for films exposed to UV-C for 13 min (film formulation RF-13min UV-C).

### 3.5. Apparent Release Kinetics of Bioactive Compounds (In Vitro)

**Figure 3:**
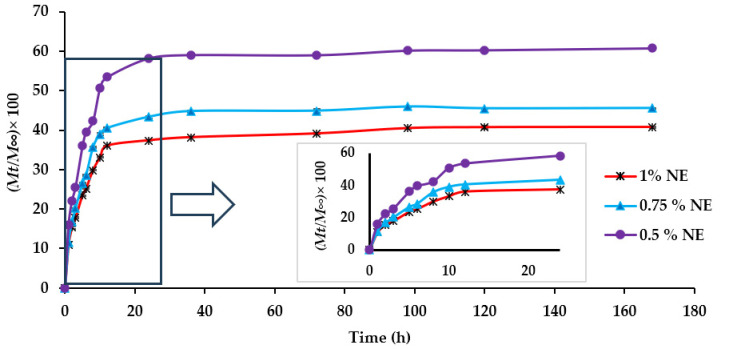
Release profile of the bioactive compounds from NE incorporated in crosslinked RF-13 min UV-C films.

The profiles of bioactive compounds released from the photo-crosslinked films in 95% ethanol during 168 h (7 days) at 4 °C are presented in Figure 3. The findings indicate that the release of bioactive compounds (EOs and citrus extract) was slow during the first 3 h and showed 17.7%, 20.1%, and 25.5% of cumulative release for 1%, 0.75%, and 0.5% NE, respectively. In comparison to 0.5% NE, the initial burst release for 1% and 0.75% NE was observed at 10 h, which was about 33.0% and 39.0% respectively; after that, a sustained release was perceived. In general, the initial burst release of the bioactive compounds is related to those situated in the outermost layer of the polymeric matrix [65]. A stabilization was observed after 12 h for 1% NE and 0.75% NE, showing a respective release of 35.9 and 40.4%. On the contrary, a release of 58.2% was observed for 0.5% NE after 24 h. So, the impacts of various concentrations of bioactive compounds on their sustained release from each film were significant (*p* ≤ 0.05). It was observed that increasing the bioactive compound concentration to 1% causes a lower cumulative release (*M_t_*/*M_∞_* × 100) compared to 0.75% and 0.5% NE (*p* ≤ 0.05). These results align with the observations made by Razavi et al. [66], who noted that in a fish GT-bacterial cellulose nanocrystal complex, higher concentrations of cinnamon EO corresponded to a decreased release rate of EO.

To analyze the release kinetics of three different concentrations of NE, the results were fitted to the Korsmeyer–Peppas model, and the parameters *k_kp_*, *n*, and *R*^2^ values are presented in Table 3. The *R*^2^ value for all film samples was > 0.9, indicating that the model fitted the release of bioactive compounds from active films. Based on the calculated *n* values for the tested samples using this model (*n* ≤ 0.45), the release was controlled by a Fickian diffusion. This suggests that the polymer remained intact throughout the release. In addition, the *k_kp_* value was inversely proportional to the concentration of bioactive compounds. For example, *k_kp_* decreased from 0.45 to 0.39 as bioactive components in the film increased from 0.5% to 1%. Thus, a higher concentration (1%) of bioactive compounds led to a decreased cumulative release over a longer period. Similarly, Criado et al. [67] observed *k_kp_* decreasing from 14.30 to 9.85 with 1–3% thyme EO in alginate beads with *n* values lower than 0.45, indicating Fickian-controlled diffusion. Ben-Fadhel et al. [36] also noted a slow Fickian diffusion-controlled release of phenolic compounds from pectin-based emulsion. Thus, these findings may demonstrate the sustained release of bioactive compounds by a Fickian diffusion from the photo-crosslinked films, which could be applied as a controlled-release active packaging system in food.

### 3.6. In Vitro Antibacterial Capacity of Bioactive Films

**Figure 4:**
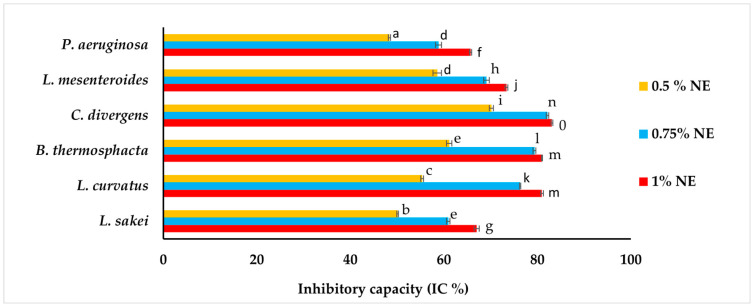
Inhibitory capacity (IC %) of crosslinked RF-13min UV-C films incorporated with 3 different concentrations of NE (0.5, 0.75, and 1%). Means followed by the same lowercase letter are not significantly different (*p* > 0.05).

The antimicrobial activity of the crosslinked film RF-13 min UV-C incorporated with three different concentrations of NE (0.5, 0.75, 1%) was tested against six spoilage bacteria (*L. sakei*, *L. curvatus*, *C. divergens*, *L. mesenteroides*, *B. thermosphacta*, and *P. aeruginosa*) using the disk diffusion method. The results of measurements of IC % are presented in Figure 4. As expected, the RF-13min UV-C film without NE (control) did not show any antibacterial activity, but bioactive films containing 0.5, 0.75, and 1% NE showed discrete antibacterial activity depending on the bacteria species (from 48.3% to 83.1%). The film incorporated with 1% NE exhibited the highest antibacterial activity (*p* ≤ 0.05), with IC values from 65.7% (against *P. aeruginosa*) to 83.1% (against *C. divergens*) compared to the two other films with 0.75% and 0.5% NE. However, films containing 0.75% and 0.5% NE showed very acceptable IC values, with a range of IC (%) extending from 48.4 to 82.2% against spoilage bacteria. It was observed that bioactive films had a significantly (*p* ≤ 0.05) higher inhibition effect on *B. thermosphacta* and *C. divergens* (61.1–80.9% and 70.1–83.1% respectively); on the contrary, the least-sensitive bacteria were *P. aeruginosa* and *L. sakei* (48.3–65.7% and 50.1–67%, respectively). The diverse antibacterial action of NE could be due to the type and morphological differences of these bacteria.

Gram (-) bacteria, such as *P. aeruginosa*, have a complex cell wall structure that limits the permeability of lipophilic compounds like EOs [68,69]. The fine and small particles of the NE—containing phenolic terpenes (thymol, carvacrol), terpene hydrocarbons (γ-terpinene, p-cymene, and α-pinene), terpenoid aldehydes (cuminic aldehyde), and phenylpropanoids (cinnamaldehyde)—exert their antimicrobial effects by disrupting bacterial cell membranes and interfering with cellular energy processes. The synergistic action of both major and minor compounds can collectively exert a substantial influence on microorganisms [70]. In our previous research, NE had the smallest particle size and polydispersity index (PDI), as well as high zeta potential and encapsulation efficiency. The prepared NE showed significantly better stability at both 4 °C and 30 °C, remaining stable for 30 days, whereas the coarse emulsion only remained stable for 5 days. Additionally, the NE demonstrated stronger antimicrobial properties, with lower minimum inhibitory concentration (MIC) against the target bacterial strains, which were not observed with the coarse emulsion [32].

### 3.7. In Situ Antimicrobial Effect of Bioactive Films—Shelf-Life of Meat

#### 3.7.1. Effect of Bioactive Films on Air-Packaged Beef

**Figure 5:**
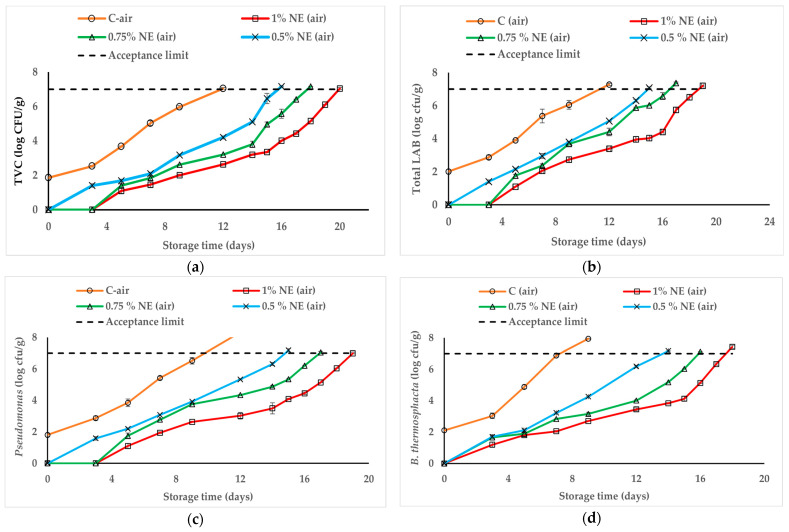
Effect of bioactive films on the microbial populations of beef stored at 4 °C in air packaging. Total viable count (TVC) (**a**), Total LAB (**b**), *Pseudomonas* spp. (**c**), and *B. thermosphacta* (**d**). The horizontal dash-dotted line represents an acceptance limit of 7 log CFU/g. Data are shown as mean ± standard deviation of 3 independent measurements.

The crosslinked film RF-13min UV-C incorporated with three different concentrations of NE (0.5, 0.75, 1%) was applied to fresh sliced beef, under air conditions. The results of microbiological analysis during storage at 4 °C, containing total viable count (TVC), total LAB count, *Pseudomonas* spp. count (PSC), and *B. thermosphacta* count (BTC), are shown in Figure 5a–d. When compared to C-air (control, meat covered with film without NE), the bioactive films exhibited significant inhibitory effects (*p* ≤ 0.05) against the studied bacteria, with similar trends in the growth curves. The initial population of TVC in C-air was 2.5 log CFU/g (Figure 5a) and eventually increased rapidly to reach 7.0 log CFU/g at day 12 (acceptance microbiological limit). The lowest TVC was found in 1% NE-air and 0.75% NE-air groups, with respective counts of 2.6 and 3.2 log CFU/g on day 12, indicating a significant difference (*p* ≤ 0.05) between these treatments and the control. Thereafter, 1% NE-air and 0.75% NE-air samples reached 7.0 log on days 20 and 18, showing a respective shelf-life extension of 8 and 6 days. In contrast, the TVC in the 0.5% NE-air group reached 7.0 log at day 15, showing a shorter shelf-life extension of 4 days. Likewise, Azarifar et al. [71] reported that the TVC of beef samples wrapped in GT-carboxymethyl cellulose films containing phenolic monoterpenes remained below the microbial limit over 15 days of storage.

LAB and *Pseudomonas* spp. are key contributors to spoilage in meat products. These bacteria can lead to significant acidification, green discoloration, the development of unpleasant flavors, and slime formation [72,73]. Based on our present findings, the initial LAB in C-air was 2.0 log CFU/g, which reached up to 7.0 log on day 12 (Figure 5b). In comparison to C-air, the LAB count in 1% NE-air and 0.75% NE-air groups was respectively 3.4 and 4.4 log on day 12 and reached 7.0 log on days 19 and 17. Hence, these bioactive films prolonged the shelf-life of meat up to 17–19 days of storage, which corresponds to a 5–7-day extension compared to C-air. As expected, the 0.5% NE-air film containing a lower percentage of NE showed a lower inhibitory effect on LAB compared to the other bioactive films, with a shelf-life extension of 3 days. Tsironi et al. [74] reported the same behavior for whey protein isolate films containing 1% EO, which had lower levels of TVC, LAB, *Pseudomonas* spp., *B. thermosphacta*, *Enterobacteriaceae*, and yeasts on the 11th day of meat shelf-life compared to films with 0.5% EOs. Very similar trends were observed against *Pseudomonas* spp., with 7 log CFU/g reached at day 12 for C-air (Figure 5c) and a significant shelf-life extension (*p* ≤ 0.05) of meat treated with 1% NE-air and 0.75% NE-air, which reached 7 log on days 19 and 17, respectively (5–7 day extension). Furthermore, the shelf-life of meat treated with 0.5% NE-air was extended until day 15 (improvement of 3 days). Generally, *Pseudomonas* is known as one of the most resistant spoilage bacteria to several antibacterial agents [75] but, in our study, bioactive films were very effective against this Gram (-) bacteria. Previous investigations by researchers also reported similar results, mentioning that films and coatings incorporated with various EOs could effectively reduce the proliferation of *Pseudomonas* in refrigerated meat [76,77,78].

*B. thermosphacta* belongs to the natural microflora of beef and is one of the predominant spoilage bacteria, which can develop a sour–sweet odor [79]. In the C-air group, the initial BTC was 2.1 log CFU/g and reached the acceptance limit (7 log) on day 7 (Figure 5d). In contrast, on day 9, a significant difference (*p* ≤ 0.05) was observed in all groups with bioactive films in descending order of 1% NE-air (2.71 log) > 0.75% NE-air (3.16 log) > 0.5% NE-air (4.26 log). The highest inhibition effect was observed for 1% NE-air, which generated an important shelf-life extension of 11 days compared to C-air. Similarly, Tsironi et al. [74] reported that the level of *B. thermosphacta* remained very low in meat products treated with whey protein-based films containing 1% EOs compared to 0.5%. Since 1% NE-air film showed a significant extension (*p* ≤ 0.05) of beef shelf-life during storage at 4 °C, this film was selected for the next study in combination with three different MAP conditions.

#### 3.7.2. Combined Effects of the Most Efficient Bioactive Film (1% NE) with MAP on Packaged Beef

**Figure 6:**
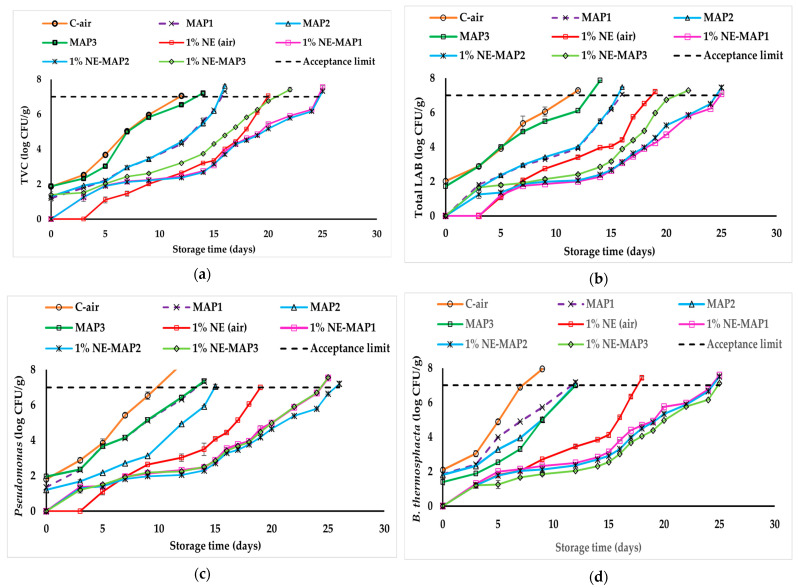
Combined effects of bioactive films and varying MAP conditions (MAP1—80% O_2_ + 20% CO_2_; MAP2—70% O_2_ + 30% CO_2_; MAP3—60% O_2_ + 20% CO_2_ + 20% N_2_) on the microbial populations of beef stored at 4 °C. Total viable count (TVC) (**a**), Total LAB (**b**), *Pseudomonas* spp. (**c**), and *B. thermosphacta* (**d**). The horizontal dash-dotted line represents an acceptance limit of 7 log CFU/g. Data are shown as mean ± standard deviation of 3 independent measurements.

The antimicrobial effects of the most efficient bioactive film (1% NE) in combination with different MAP conditions consisting of 80% O_2_ + 20% CO_2_ (MAP1), 70% O_2_ + 30% CO_2_ (MAP2), and 60% O_2_ + 20% CO_2_ + 20% N_2_ (MAP3) were evaluated on beef samples for 26 days at 4 °C, and data are reported in Figure 6a–d. As observed in the previous section, when compared to C-air (control, meat covered with film without NE), the bioactive films exhibited significant inhibitory effects (*p* ≤ 0.05) against the studied bacteria, with similar zone trends in the growth curves. In all bacterial counts, the initial level of control (C-air) was determined to be 2 log CFU/g on day 0. The TVC level reached 7 log CFU/g on different days depending on the sample groups (Figure 6a). In ascending order, the following measurements were obtained: day 12 (C-air) < day 14 (MAP3) < day 16 (MAP1 and MAP2) < day 20 (1% NE-air) < day 22 (1% NE-MAP3) < day 25 (1% NE-MAP1 and 1% NE-MAP2), with significant differences (*p* ≤ 0.05). Therefore, the sequence of shelf-life extension followed the same influence of parameters in the order air < MAP3 < MAP1 (or MAP2) < bioactive film < bioactive film + MAP3 < bioactive film + MAP1 (or +MAP2). Thus, the single MAP process allowed a significant shelf-life extension (*p* ≤ 0.05) between 2 and 4 days, with a lower impact by using MAP3 (low O_2_ content compared to MAP1 and MAP2). Furthermore, the combination of bioactive film with high-O_2_ MAP (MAP1 or MAP2) provided the highest TVC inhibition, extending the shelf-life of beef by 13 days in comparison to C-air (more than double). Huang et al. [80] also noted a lower TVC in meat products at 60% O_2_ compared to 20% and 40% O_2_ MAP. The present results align reasonably well with those of Berruga et al. [81], who observed a TVC exceeding 7 log CFU/g in lamb meat stored for 3 to 4 weeks using different MAP conditions. Similarly, Xiong et al. [82] found that an oregano–resveratrol NE/pectin coating on pork loins under high-O_2_ MAP resulted in the lowest TVC values throughout 20 days of storage.

As described for the results obtained in air-packaged meat, very similar graphs were observed between total LAB (Figure 6b) and *Pseudomonas* spp. (Figure 6c), except an inversion of the efficiency between MAP1 and MAP2 as single or combined treatments. Indeed, in both graphs (Figure 6c), control groups (C-air) reach 7.0 log CFU/g at day 12, and successive significant shelf-life extensions (*p* ≤ 0.05) are observed in meat treated with the different processes. For total LAB (Figure 6a), the extension follows the ascending order day 12 (C-air) < day 14 (MAP3) < day 16 (MAP1 and MAP2) < day 19 (1% NE-air) < day 22 (1% NE-MAP3) < day 25 (1% NE-MAP1 and 1% NE-MAP2), which is also very similar to the trend observed in TVC. In comparison, for *Pseudomonas*, the extension follows the ascending order day 12 (C-air) < day 14 (MAP3 and MAP1) < day 15 (MAP2) < day 19 (1% NE-air) < day 24 (1% NE-MAP3 and 1% NE-MAP1) < day 26 (1% NE-MAP2), resulting in a lower efficiency of MAP1 process.

Regarding LAB count (Figure 6b), there was no significant difference (*p* > 0.05) between MAP1 and MAP2 throughout storage, both reaching 7 log on day 16 (4-day extension), while MAP3 reached 7.0 log on day 14 (2-day extension only). Otherwise, the combined treatments with bioactive film 1% NE-MAP1 and 1%NE-MAP2 reached 7.0 log on day 25 (compared to day 22 for 1% NE-MAP3), corresponding to a shelf-life extension of 13 days (more than double) and an extension of 6 to 9 days compared to their respective single treatments. Hence, 1% NE-MAP1 and 1% NE-MAP2 were the most efficient treatments for controlling the LAB population in beef. It is clear from Figure 6b that the contribution of MAP1 had a higher inhibitory effect on LAB populations, due to the fact they are facultative anaerobic bacteria. LAB levels remain consistently low in high-O_2_ MAP (70–80% O_2_) and have a limited impact on the spoilage microflora [83]. According to Jaberi et al. [84], vacuum-packaged buffalo meat showed faster growth of LAB than high-O_2_ MAP (80% O_2_ + 20% CO_2_). Berruga et al. [81] also reported an increase in LAB count in vacuum-packaged ground beef compared to high-O_2_ MAP samples during storage.

Regarding *Pseudomonas* spp. (Figure 6c), it is interesting to note such an inversion of the effect of MAP2 to the detriment of MAP1. Indeed, no significant difference (*p* ˃ 0.05) was observed between MAP1 and MAP3 throughout storage, both reaching 7 log on day 14 (2-day extension only), while MAP2 reached 7.0 log on day 15 (3-day extension). Otherwise, the combined treatment with bioactive film 1% NE-MAP2 reached 7.0 log on day 26 (compared to day 24 for 1% NE-MAP1 and 1% NE-MAP3), corresponding to a shelf-life extension of 14 days (more than double) and an extension of 7 to 11 days compared to its respective single treatments. Hence, 1% NE-MAP2 was the most efficient treatment for controlling *Pseudomonas* in beef. This inverted tendency can be explained since *Pseudomonas* spp. are Gram (-) bacteria and they are notably sensitive to CO_2_, which prolongs their lag phase and slows their growth rate throughout the logarithmic phase [85]. Using 20% or more CO_2_ in MAP significantly inhibits their growth [86]. Thus, MAP1, MAP2, and MAP3 showed greater inhibition as compared to C-air (Figure 6c), and higher CO_2_ concentrations in the MAP gas mixtures resulted in more significant *Pseudomonas* inhibition (*p* ≤ 0.05). Also, their combination with active film effectively inhibited *Pseudomonas* spp. in fresh beef. This aligns with findings by Chouliara et al. [5] on the high inhibition effects of MAP combined with 1% oregano EO. Our study confirms that MAP2, with higher CO_2_ concentration, was more effective in reducing *Pseudomonas* spp. This supports the finding that these bacteria are more susceptible to CO_2_ compared to Gram (+) bacteria such as LAB and *B. thermosphacta* [87,88].

*B. thermosphacta,* a Gram (+) facultative anaerobe bacterium, is included in the natural microflora of fresh meat that is packaged either in aerobic conditions or under MAP [89]. This could explain the similar trend of increasing shelf-life with a lower efficiency of MAP1, as observed in *Pseudomonas*. Indeed, the extension follows the ascending order (Figure 6d) day 7 (C-air) < day 12 (MAP1, MAP2 and MAP3) < day 18 (1% NE-air) < day 24 (1% NE-MAP1 and 1% NE-MAP2) < day 25 (1% NE-MAP3). As single treatments, MAP1, MAP2, and MAP3 generated an intermediate zone with significant differences (*p* ≤ 0.05) during storage but with a common terminal point of 7 log CFU/g at day 12 (*p* > 0.05), corresponding to a 5-day extension. Thus, during storage, MAP2 and MAP3 were more efficient than MAP1 at inhibiting bacterial count. This tendency was maintained when MAP was combined with bioactive film. Specifically, the combined treatment of 1% NE-MAP3 reached 7.0 log on day 25 (compared to day 24 for 1% NE-MAP1 and 1% NE-MAP2), corresponding to a shelf-life extension of 18 days (more than 3 times), and an extension of 7 to 13 days compared to its respective single treatments. Hence, 1% NE-MAP3 was the most efficient treatment for controlling *B. thermosphacta* in beef. This competitive bacterium grows more rapidly when oxygen is available for aerobic metabolism [90]. These results also align with Chouliara et al. [5], who observed a decrease of over 5 logs in *B. thermosphacta* when using a combination of MAP and oregano EO compared to individual treatment during 25 days of storage.

In summary, the data obtained in our in situ microbiological study confirm a synergistic effect between bioactive film and MAP, with a MAP process to delay TVC, LAB growth, and spoilage bacteria such as *Pseudomonas* spp. and *B. thermosphacta* in meat, depending on the O_2_ and CO_2_ content. It was also reported that NEs can improve the dispersion of antimicrobial agents in aqueous phase or food matrices, efficiently transport through outer membrane porin proteins, and deliver EOs to bacterial cell membranes [91], thereby providing a strong antimicrobial effect and a longer shelf-life of products (26 days of storage in our study). Masoomian et al. [42] found that combining nanoencapsulated EOs and high-O_2_ MAP (80% O_2_ + 20% CO_2_) effectively enhanced the microbial and sensory quality of ground meat stored at 4 °C.

### 3.8. Degree of Lipid Oxidation in Meat (TBARS Measurement)

The TBARS value is a common measure for lipid oxidation in meat, determined by the red pigment produced when 2-thiobarbituric acid reacts with malondialdehyde (MDA), a by-product of polyunsaturated fatty acid oxidation [92]. The permissible limit of TBARS values for the acceptability of beef is 2.0 mg MDA/kg [93]. Table 4 shows the TBARS values of samples during storage at 4 °C, in parallel with microbiological analyses.

On day 0, TBARS values were 0.03–0.04 mg MDA/kg among all groups (*p* > 0.05), which increased over time. Notably, the control group (C-air) exceeded the limit on day 9 and reached 3.27 mg MDA/kg on day 12, indicating the highest degree of lipid oxidation compared to other samples. However, on day 9, MAP1, MAP2, and MAP3 treatments generated 0.68, 0.63, and 0.41 mg MDA/kg, respectively, showing a significant reduction compared to the levels found in C-air (*p* ≤ 0.05). Among MAP groups, TBARS values were particularly affected by oxygen levels in MAP composition, with values increasing significantly (*p* ≤ 0.05) with oxygen content, as follows: 0.41 mg MDA/kg at 60% O_2_ (MAP3) < 0.63 mg MDA/kg at 70% O_2_ (MAP2) < 0.68 mg MDA/kg at 80% O_2_ (MAP1). Both MAP1 and MAP2 exceeded the limit on day 16, while MAP3 remained below the limit at 1.60 mg MDA/kg (*p* ≤ 0.05). As reported by Śmiecińska and Daszkiewicz [92], the most significant oxidative changes in lipids occurred in beef samples stored with high oxygen levels (80% O_2_). In contrast, the combined treatment of active film with different MAP conditions greatly retarded the lipid oxidation of beef, with TBARS values much lower (*p* ≤ 0.05) than those of the control group (C-air), remaining below the limit during 26 days of storage in a range of 1.12–1.37 mg MDA/kg. No notable variation (*p* > 0.05) was observed between 1% NE-MAP1 and 1% NE-MAP2 samples throughout storage (*p* ˃ 0.05), whereas 1% NE-MAP3 was significantly lower (*p* ≤ 0.05) from day 14. The bioactive film (1% NE) played a crucial role in reducing lipid oxidation in the combined treatments, as it maintained significantly low TBARS values (≤0.53 mg MDA/kg) throughout the storage period compared to single MAP treatments (≥1 mg MDA/kg from day 14). Hence, the potent antioxidant effects of the bioactive films are likely due to the abundant flavonoid compounds and phenol terpenes present in the NE formulation. Phenolic and flavonoid compounds found in citrus extract and EOs prevent oxidation by donating electrons, disrupting propagation steps, scavenging free radicals, binding metal ions, or serving as substrates for radicals like superoxide or hydroxyl [82,94]. Consistent with our findings, Xiong et al. [82] demonstrated that incorporating oregano EO and/or resveratrol into a pectin coating enhanced the ability of pork samples under high-O2 MAP (80% O_2_ + 20% CO_2_) to resist lipid oxidation.

### 3.9. Meat Color Assessment

The meat color was also evaluated in parallel with the microbiological analyses, for 26 days storage at 4 °C. The color properties, which consist of lightness (L*), redness (a*), and yellowness (b*), are detailed in Table 5. L* typically increased in all samples, possibly due to the oxidation of myoglobin in meat. The initial L* values of all groups (at day 0) ranged between 40.3 and 40.7 (*p* ˃ 0.05). Table 5 shows that as storage time increased, the L* values of the control group (C-air) rose quickly, while those of the bioactive film alone and the combination of film with MAP remained more stable. Indeed, L* increased from 40.5 to 55.5 after 12 days, whereas it only increased from 40.2–40.7 to 43.1–45.8. Additionally, on day 16, samples with active film and active film combined with MAP had significantly lower L* values (*p* ≤ 0.05) compared to single MAP treatments. Indeed, L* values were 48.8–50.2 for MAP treatments, 45.7 for the bioactive film (1% NE), and 44.0–44.2 for combined treatments. Overall, from day 16 to day 26, the lowest L* values were observed for the combined treatments, with no significant differences (*p* ˃ 0.05) between them. These findings suggest that beef became slightly paler as it deteriorated, but the combined process of active film and MAP could effectively prevent this evolution.

Redness (a*) is a key indicator for customers to judge meat freshness, reflecting the oxygenation status of myoglobin. The initial a* values of all groups (at day 0) ranged from 22.3 to 22.8 (*p* > 0.05). C-air samples showed a rapid decline in a* values from 22.4 (day 0) to 3.0 (day 12), indicating very low oxymyoglobin levels on the meat surface. In contrast, MAP1 and MAP2 maintained initial a* values until day 5 (*p* > 0.05), whereas MAP3 did so only until day 3. On day 12, a* values of MAP1, MAP2, and MAP3 were 15.5, 16.2, and 16.5, respectively, whereas significantly higher values (*p* ≤ 0.05) were successively obtained with 1% NE (19.8 in air-packaged beef with bioactive film), as well as in the combined treatments with MAP2 or MAP3 (21.2–21.4) and the combined treatments with MAP1 (23.0). The latter process (bioactive film combined with MAP1) allowed (*p* > 0.05) the redness of the meat to be maintained during these 12 days of storage and until day 17, with a significantly higher value (*p* ≤ 0.05) compared to the two other combined treatments with MAP2 and MAP3. Then, after day 12, the redness of meat decreased continuously with combined treatments, but at satisfying degrees until day 19 (a* > 15).

Hence, high-O_2_ MAP (MAP1 and MAP2) promoted oxymyoglobin (bright red color) formation. Alternatively, it must be noted that bioactive film under air (1% NE) retained redness throughout storage, with significantly higher values (*p* ≤ 0.05) than those of single MAP treatments, which could indicate its contribution in combined treatments. Holman et al. [95] suggested beef color is acceptable when a* is ≥ 14.5. The best results obtained for the combination of bioactive film with MAP showed a significant (*p* ≤ 0.05) protective effect on meat redness, likely due to the antioxidant effects of bioactive compounds in the NE system. Interestingly, the 1% NE-MAP1 sample increased the redness of meat from day 0 to day 7; then, redness decreased slowly compared to other treatments. This initial increase could be due to myoglobin pigments’ exposure to high O_2_ concentration, converting deoxymyoglobin (purple) to oxymyoglobin [96]. Thus, the combination of active film plus MAP1 synergistically retained beef redness. Similar results were found for pork coated with EO-based NE under high-O_2_ MAP (80% O_2_ + 20% CO_2_) [82].

The yellowness (b*) of all beef samples increased over time, aligning with the results reported by Karabagias et al. [97] and Berruga et al. [81], who noted a rise in L* and b* values in lamb meat as time progressed. In the present study, the control group (C-air) displayed a significant and rapid increase in b* (from 15.4 to 20.4) (*p* ≤ 0.05) until day 12 compared to other samples. This change may be attributed to lipid oxidation during storage, as higher b* values are linked to greater lipid oxidation [98], and the resulting accumulation of brown metmyoglobin [99]. On day 12, there was a notable difference in b* values between the MAP treatments and C-air (*p* ≤ 0.05). This difference could be due to the increased oxygen content in MAP-packaged beef, leading to oxymyoglobin and a redder appearance compared to air-packaged beef. Conversely, the 1% NE sample showed a slower increase in b* values compared to the MAP groups (*p* ≤ 0.05). Additionally, the 1% NE-MAP samples had the lowest b* value in comparison to the 1% NE, MAP, and C-air samples. The reduction in metmyoglobin formation seems to result from the combined effects of the bioactive film and MAP, as shown by higher a* and lower b* values. Finally, the combination of NE with various MAP conditions had a beneficial and synergistic effect on the color parameters of beef, particularly the redness a*, which reached high levels and remained stable for extended storage duration.

## 4. Conclusions

Our study found that utilizing UV-C radiation for 13 min to crosslink GT in the presence of RF was an efficient and eco-friendly method of biobased film production. This process allowed the creation of a film with highly improved physicochemical properties by overcoming the hydrophilic limitations of such a biopolymer-based material. RF, at low concentrations, served as a photosensitizer for an innovative crosslinking method, enhancing mechanical strength, reducing moisture permeability, and decreasing solubility in water of GT-based films. FTIR analysis demonstrated that the crosslinking process occurred via conformational changes in the protein network and the appearance of new bands related to imidazolone groups involved in RF-GT covalent linkage. This green technology could replace common chemical crosslinking agents. The 1% NE-incorporated bioactive film showed a significant extension of beef shelf-life during storage at 4 °C. The main factor contributing to the antimicrobial effectiveness is the capacity of the crosslinked film to retain bioactive compounds and release them in a controlled manner over time. In vitro release study also showed that the bioactive film containing 1% NE had the highest controlled diffusion and lowest release rate of bioactive compounds compared to 0.75% and 0.5% NE. The combination of 1% NE bioactive film with different MAP compositions further extended the shelf-life of beef, with high-O_2_ MAP (70–80% O_2_) providing the best preservation and maintaining meat color, especially the redness (a*). These combined treatments not only maintained and prolonged meat redness, but also prevented lipid oxidation (< 2 mg MDA/kg) during storage. Thus, the bioactive film combined with high-O_2_ MAP proved to be the best treatment for prolonging the shelf-life of fresh sliced beef.

## Figures and Tables

**Table 1:** Effect of different RF concentrations (% *w*/*w* of GT) on the mechanical properties (TS, TM, and Eb) of GT-based films.

Films	TS (MPa)	TM (MPa)	Eb (%)
Pure GT (control)	21.02 ± 0.33 ^a^	125.93 ± 1.66 ^a^	86.01 ± 4.25 ^d^
0.3% RF	21.66 ± 1.45 ^a^	125.27 ± 1.69 ^a^	74.37 ± 2.63 ^b^
0.5% RF	22.85 ± 0.56 ^ab^	216.07 ± 4.39 ^b^	64.25 ± 3.99 ^a^
0.75% RF	24.74 ± 1.41 ^b^	278.49 ± 2.39 ^c^	67.62 ± 1.08 ^a^
1.25% RF	28.05 ± 1.24 ^c^	294.49 ± 2.10 ^d^	80.55 ± 1.48 ^c^

TS: Tensile Strength; TM: Tensile Modulus; Eb: Elongation at Break. Values are presented as mean ± standard deviation. In each column, means with the same lowercase letter are not significantly different (*p* > 0.05).

**Table 2:** Physico-mechanical properties of UV-C-irradiated crosslinked GT-based films.

Films	TS (MPa)	TM (MPa)	Eb (%)	WVP (g·mm/m^2^·day·kPa)	WS (%)
Pure GT (without RF) without UV-C	21.02 ± 0.33 ^b^	125.93 ± 1.66 ^a^	86.01 ± 4.25 ^e^	20.49 ± 0.29 ^i^	100 ± 0.00
RF without UV-C	28.05 ± 1.24 ^c^	294.49 ± 2.10 ^c^	80.55 ± 1.48 ^de^	17.11 ± 0.14 ^h^	31.78 ± 0.36 ^i^
RF-4 min UV-C	34.14 ± 0.9 ^d^	323.51 ± 2.56 ^d^	82.83 ± 6.73 ^e^	13.33 ± 0.18 ^f^	22.14 ± 1.74 ^g^
RF-9 min UV-C	44.11 ± 4.57 ^fg^	427.6 ± 2.13 ^g^	35.26 ± 0.94 ^a^	9.72 ± 0.15 ^e^	17.38 ± 0.74 ^f^
RF-13 min UV-C	46.69 ± 2.18 ^g^	573.1 ± 2.43 ^j^	67.4 ± 1.98 ^c^	6.71 ± 0.18 ^c^	11.85 ± 0.083 ^d^
RF-17 min UV-C	42.35 ± 3.05 ^ef^	495.06 ± 8.52 ^i^	74.43 ± 5.09 ^cd^	7.42 ± 0.23 ^d^	13.08 ± 0.069 ^e^
RF-22 min UV-C	9.33 ± 0.77 ^a^	166.4 ± 3.8 ^b^	44.6 ± 5.28 ^b^	14.74 ± 0.39 ^g^	25.26 ± 0.61 ^h^
RF-0.5% NE-13 min UV-C	43.31 ± 0.53 ^fg^	470.59 ± 5.79 ^h^	70.61 ± 2.43 ^c^	6.27 ± 0.45 ^c^	9.37 ± 0.24 ^c^
RF-0.75% NE-13 min UV-C	40.69 ± 2.19 ^ef^	410.59 ± 10.23 ^f^	81.95 ± 3.05 ^e^	4.87 ± 0.72 ^b^	6.82 ± 0.51 ^b^
RF-1% NE-13 min UV-C	38.78 ± 1.46 ^e^	333.92 ± 7.39 ^e^	97.99 ± 3.51 ^f^	3.74 ± 0.32 ^a^	5.50 ± 0.24 ^a^

Values are presented as mean ± standard deviation. In each column, means with the same lowercase letter are not significantly different (*p* > 0.05). RF was used at a concentration of 1.25% (*w*/*w* of GT).

**Table 3:** Release parameters of the bioactive compounds from NE incorporated in crosslinked RF-13min UV-C films.

Samples	*n*	*k_kp_*	*R* ^2^
1% NE	0.42	0.390	0.9625
0.75% NE	0.45	0.404	0.9579
0.5% NE	0.44	0.459	0.9672

Parameters *n*, *k_kp_*, and *R*^2^ represent release exponent, release constant, and correlation coefficient, respectively. Data are shown as mean ± standard deviation of 3 independent measurements.

**Table 4:** TBARS content of beef packaged with bioactive film alone and in combination with varying MAP conditions.

Attribute	Samples	Days																
		0	3	5	7	9	12	14	15	16	17	18	19	20	22	24	25	26
TBARS	C-air	0.04 ± 0.0 ^bA^	0.20 ± 0.03 ^dB^	0.50 ± 0.02 ^eC^	0.70 ± 0.01 ^gD^	2.14 ± 0.04 ^gE^	3.27 ± 0.24 ^dF^	-	-	-	-	-	-	-	-	-	-	-
	MAP1	0.03 ± 0.0 ^aA^	0.16 ± 0.01 ^cB^	0.32 ± 0.03 ^dC^	0.54 ± 0.02 ^fD^	0.68 ± 0.01 ^fE^	0.89 ± 0.03 ^cF^	1.01 ± 0.02 ^dG^	1.95 ± 0.04 ^eH^	2.44 ± 0.1 ^eI^	-	-	-	-	-	-	-	-
	MAP2	0.03 ± 0.0 ^aA^	0.16 ± 0.01 ^cB^	0.30 ± 0.02 ^dC^	0.51 ± 0.03 ^eD^	0.63 ± 0.03 ^eE^	0.85 ± 0.02 ^cF^	1.01 ± 0.01 ^dG^	1.86 ± 0.01 ^dH^	2.20 ± 0.07 ^dI^	-	-	-	-	-	-	-	-
	MAP3	0.03 ± 0.0 ^aA^	0.08 ± 0.0 ^bA^	0.22 ± 0.01 ^cB^	0.35 ± 0.02 ^dC^	0.41 ± 0.02 ^dC^	0.60 ± 0.04 ^bD^	0.94 ± 0.05 ^cE^	1.05 ± 0.03 ^cF^	1.60 ± 0.06 ^cG^	-	-	-	-	-	-	-	-
	1% NE	0.04 ± 0.0 ^aA^	0.03 ± 0.0 ^aA^	0.04 ± 0.01 ^aA^	0.05 ± 0.01 ^aAB^	0.08 ± 0.01 ^aBC^	0.11 ± 0.01 ^aCD^	0.14 ± 0.01 ^aD^	0.18 ± 0.02 ^aE^	0.23 ± 0.02 ^aF^	0.30 ± 0.01 ^aG^	0.37 ± 0.02 ^bH^	0.48 ± 0.05 ^bI^	0.53 ± 0.05 ^bJ^	-	-	-	-
	1% NE-MAP1	0.03 ± 0.0 ^aA^	0.05 ± 0.0 ^aA^	0.07 ± 0.01 ^bA^	0.14 ± 0.0 ^cB^	0.17 ± 0.01 ^cBC^	0.19 ± 0.01 ^aC^	0.22 ± 0.01 ^bC^	0.27 ± 0.02 ^bD^	0.35 ± 0.03 ^bE^	0.54 ± 0.03 ^bF^	0.58 ± 0.01 ^cFG^	0.61 ± 0.01 ^cGH^	0.65 ± 0.02 ^cH^	0.72 ± 0.01 ^bI^	0.92 ± 0.05 ^bJ^	1.21 ± 0.05 ^bK^	1.37 ± 0.08 ^bL^
	1% NE-MAP2	0.03 ± 0.0 ^aA^	0.04 ± 0.0 ^aA^	0.07 ± 0.0 ^bA^	0.13 ± 0.01 ^cB^	0.16 ± 0.01 ^cBC^	0.19 ± 0.01 ^aC^	0.21 ± 0.01 ^bC^	0.25 ± 0.01 ^bD^	0.33 ± 0.06 ^bE^	0.51 ± 0.02 ^bF^	0.56 ± 0.01 ^cG^	0.59 ± 0 ^cG^	0.63 ± 0.02 ^cH^	0.71 ± 0 ^bI^	0.92 ± 0.04 ^bJ^	1.20 ± 0.04 ^bK^	1.34 ± 0.06 ^bL^
	1% NE-MAP3	0.03 ± 0.0 ^aA^	0.03 ± 0.0 ^aA^	0.06 ± 0.01 ^bAB^	0.08 ± 0.01b ^ABC^	0.12 ± 0.01b ^BCD^	0.14 ± 0.01 ^aCD^	0.16 ± 0.01 ^aDE^	0.17 ± 0.01 ^aDE^	0.21 ± 0.01 ^aE^	0.29 ± 0.01 ^aF^	0.32 ± 0.01 ^aF^	0.38 ± 0.01 ^aG^	0.41 ± 0.01 ^aG^	0.54 ± 0.02 ^aH^	0.66 ± 0.03 ^aI^	0.92 ± 0.07 ^aJ^	1.12 ± 0.11 ^aK^

MAP1—80% O_2_ + 20% CO_2_; MAP2—70% O_2_ + 30% CO_2_; MAP3—60% O_2_ + 20% CO_2_ + 20% N_2_. Values are presented as mean ± standard deviation. Within each row, means with the same uppercase letter are not significantly different (*p* > 0.05). Within each column, means with the same lowercase letter are not significantly different (*p* > 0.05).

**Table 5:** Color parameters of beef packaged with bioactive film alone and in combination with varying MAP conditions.

Attribute	Samples	Days																
		0	3	5	7	9	12	14	15	16	17	18	19	20	22	24	25	26
L*	C-air	40.5 ± 0.35 ^aA^	40.9 ± 0.20 ^bB^	43.6 ± 0.15 ^dC^	46.3 ± 0.23 ^dD^	51.4 ± 0.20 ^gE^	55.5 ± 0.25 ^cF^	-	-	-	-	-	-	-	-	-	-	-
	MAP1	40.3 ± 0.25 ^aA^	40.4 ± 0.15 ^aA^	40.7 ± 0.25 ^abcA^	43.3 ± 0.15 ^cB^	44.3 ± 0.37 ^fC^	45.8 ± 0.20 ^bD^	49.4 ± 0.15 ^cE^	50.4 ± 0.25 ^eF^	50.2 ± 0.20 ^dF^	-	-	-	-	-	-	-	-
	MAP2	40.5 ± 0.60 ^aA^	40.4 ± 0.25 ^aA^	40.9 ± 0.20 ^bcA^	42.8 ± 0.25 ^cB^	43.4 ± 0.23 ^eC^	45.6 ± 0.3 ^bD^	49.2 ± 0.25 ^cE^	50.2 ± 0.15 ^eF^	50.2 ± 0.30 ^dF^	-	-	-	-	-	-	-	-
	MAP3	40.7 ± 0.45 ^aA^	40.9 ± 0.32 ^bA^	41.2 ± 0.30 ^cA^	41.2 ± 0.2 ^bA^	42.6 ± 0.1 ^dB^	43.8 ± 0.25 ^aC^	47.1 ± 0.30 ^bD^	48.6 ± 0.20 ^dE^	48.8 ± 0.47 ^cE^	-	-	-	-	-	-	-	-
	1% NE	40.2 ± 0.15 ^aAB^	40.2 ± 0.25 ^aA^	40.4 ± 0.32 ^abAB^	40.8 ± 0.72 ^abB^	42 ± 0.4 ^cC^	43.8 ± 0.3 ^aD^	44.5 ± 0.3 ^aE^	45.2 ± 0.4 ^cF^	45.7 ± 0.15 ^bF^	46.4 ± 0.2 ^bG^	46.6 ± 0.05 ^bG^	47.4 ± 0.32 ^cH^	48 ± 0.4 ^bI^	-	-	-	-
	1% NE-MAP1	40.4 ± 0.32 ^aA^	40.5 ± 0.1 ^aA^	40.8 ± 0.15 ^abcA^	40.6 ± 0.72 ^abA^	40.9 ± 0.15 ^abA^	43.5 ± 0.4 ^aB^	44.2 ± 0.36 ^aDC^	44.5 ± 0.30 ^abC^	44 ± 0.1 ^aBC^	45.3 ± 0.2 ^aD^	45.2 ± 0.20 ^aD^	45.3 ± 0.2 ^aD^	46.3 ± 0.30 ^aE^	46.5 ± 0.32 ^aE^	47.2 ± 0.45 ^aF^	47.6 ± 0.25 ^aFG^	47.9 ± 0.47 ^aG^
	1% NE-MAP2	40.6 ± 0.15 ^aA^	40.4 ± 0.35 ^aA^	40.5 ± 0.30 ^abA^	40.2 ± 0.45 ^aA^	41.4 ± 0.23 ^bB^	43.1 ± 1.01 ^aC^	44 ± 0.15 ^aD^	44.2 ± 0.26 ^aD^	44.2 ± 0.40 ^aD^	45 ± 0.15 ^aE^	45 ± 0.25 ^aE^	45 ± 0.15 ^aE^	46.2 ± 0.36 ^aF^	46.3 ± 0.37 ^aF^	47.1 ± 0.34 ^aG^	47.6 ± 0.37 ^aGH^	48 ± 0.58 ^aH^
	1% NE-MAP3	40.5 ± 0.17 ^aA^	40.5 ± 0.05 ^aA^	40.3 ± 0.49 ^aA^	40.6 ± 0.17 ^abA^	40.8 ± 0.30 ^aA^	43.1 ± 0.25 ^aB^	44.1 ± 0.20 ^aC^	44.7 ± 0.05 ^bD^	44 ± 0.43 ^aC^	44.9 ± 0.28 ^aD^	45.2 ± 0.87 ^aD^	45.8 ± 0.05 ^bE^	46 ± 0.15 ^aEF^	46.4 ± 0.41 ^aF^	47.7 ± 0.15 ^aG^	47.7 ± 0.20 ^aG^	48.3 ± 0.36 ^aG^
a*	C-air	22.4 ± 0.15 ^aF^	20.2 ± 0.36 ^aE^	17.4 ± 0.15 ^aD^	12.1 ± 0.43 ^aC^	8.1 ± 0.55 ^aB^	3 ± 0.25 ^aA^	-	-	-	-	-	-	-	-	-	-	-
	MAP1	22.7 ± 0.35 ^aG^	22.5 ± 0.30 ^bG^	22.2 ± 0.41 ^cdG^	18.5 ± 0.15 ^bF^	16.9 ± 0.87 ^bE^	15.5 ± 0.20 ^bD^	12.4 ± 0.15 ^aC^	10.2 ± 0.49 ^aB^	8.2 ± 0.30 ^aA^								
	MAP2	22.4 ± 0.20 ^aF^	22.4 ± 0.15 ^bF^	22 ± 0.49 ^cF^	18.8 ± 0.79 ^bE^	16.9 ± 0.66 ^bD^	16.2 ± 0.55 ^cD^	12.5 ± 0.35 ^aC^	10.9 ± 0.40 ^bB^	9.7 ± 0.95 ^bA^								
	MAP3	22.6 ± 0.1 ^aH^	22.6 ± 0.1 ^bH^	20.2 ± 0.30 ^bG^	19.6 ± 0.3 ^cF^	18.1 ± 0.45 ^cE^	16.5 ± 0.36 ^cD^	14.5 ± 0.3 ^bC^	12.6 ± 0.2 ^cB^	9.4 ± 0.35 ^bA^								
	1% NE	22.6 ± 0.1 ^aK^	22.4 ± 0.15 ^bK^	22.1 ± 0.05 ^cdJ^	22 ± 0.3 ^dJ^	21.2 ± 0.15 ^dI^	19.8 ± 0.36 ^dH^	17.3 ± 0.25 ^cG^	15.5 ± 0.32 ^dF^	14.3 ± 0.41 ^cE^	13.9 ± 0.7 ^aD^	11.7 ± 0.7 ^aC^	11.1 ± 0.78 ^aB^	9.6 ± 0.58 ^aA^	-	-	-	-
	1% NE-MAP1	22.6 ± 0.1 ^aI^	25.1 ± 0.30 ^dJ^	25.3 ± 0.15 ^fJ^	25.1 ± 0.30 ^fJ^	23.3 ± 0.40 ^fI^	23 ± 0.26 ^fI^	20.5 ± 0.2 ^fH^	20.6 ± 0.26 ^gH^	19.7 ± 0.66 ^fG^	19.3 ± 0.95 ^dG^	18.4 ± 0.25 ^cF^	18.1 ± 0.15 ^cF^	14.1 ± 0.30 ^bE^	12.6 ± 0.2 ^aD^	11.4 ± 0.25 ^aC^	9.8 ± 0.55 ^aB^	7.9 ± 0.52 ^aA^
	1% NE-MAP2	22.8 ± 0.56 ^aI^	23.3 ± 0.30 ^cI^	23.1 ± 0.55 ^eI^	23 ± 0.26 ^eI^	23.2 ± 0.40 ^fI^	21.2 ± 0.45 ^eH^	19.6 ± 0.56 ^eG^	19.9 ± 0.20 ^fG^	18.7 ± 0.7 ^eF^	18.2 ± 0.15 ^cF^	18.2 ± 0.15 ^cF^	18.1 ± 0.25 ^cF^	13.9 ± 0.41 ^bE^	12.6 ± 0.43 ^aD^	11.4 ± 0.26 ^aC^	9.6 ± 0.35 ^aB^	7.8 ± 0.2 ^aA^
	1% NE-MAP3	22.3 ± 0.25 ^aJ^	23.4 ± 0.2 ^cK^	22.7 ± 0.1d ^eJ^	22.3 ± 0.26 ^dJ^	22.2 ± 0.23 ^eJ^	21.4 ± 0.25 ^eI^	18.4 ± 0.2 ^dH^	18.2 ± 0.15 ^eH^	16.6 ± 0.15 ^dG^	16 ± 0.25 ^bF^	16 ± 0.25 ^bF^	16.4 ± 0.47 ^bFG^	14.6 ± 0.32 ^bE^	12.5 ± 0.20 ^aD^	11.1 ± 0.62 ^aC^	9.2 ± 0.26 ^aB^	8 ± 0.32 ^aA^
b*	C-air	15.4 ± 0.49 ^aA^	15.3 ± 0.32 ^abcA^	16.6 ± 0.2 ^bB^	18 ± 0.41 ^dC^	18.5 ± 0.25 ^cC^	20.4 ± 0.2 ^eD^											
	MAP1	15.5 ± 0.25 ^aA^	15.7 ± 0.1 ^cA^	15.5 ± 0.15 ^aA^	16 ± 0.25 ^cB^	16.6 ± 0.15 ^bC^	17.1 ± 0.20 ^dD^	17.7 ± 0.15 ^dE^	18 ± 0.1 ^eE^	18.8 ± 0.20 ^eF^								
	MAP2	15.3 ± 0.15 ^aA^	15.3 ± 0.32 ^abcA^	15.2 ± 0.30 ^aA^	15.9 ± 0.66 ^bcB^	16.5 ± 0.26 ^bC^	17.3 ± 0.15 ^dD^	17.5 ± 0.20 ^dD^	18.3 ± 0.1 ^eE^	18.5 ± 0.15 ^eE^								
	MAP3	15.5 ± 0.20 ^aA^	15.5 ± 0.1 ^bcA^	15.5 ± 0.30 ^aA^	15.3 ± 0.30 ^abcA^	15.4 ± 0.25 ^aA^	16.4 ± 0.26 ^cB^	16.7 ± 0.15 ^cB^	17.3 ± 0.20 ^dC^	17.8 ± 0.26 ^dD^								
	1% NE	15.3 ± 0.15 ^aA^	15.3 ± 0.2 ^abcA^	15.5 ± 0.2 ^aA^	15.4 ± 0.2 ^abcA^	15.6 ± 0.11 ^aAB^	15.9 ± 0.1 ^bBC^	16 ± 0.15 ^bC^	16.1 ± 0.15 ^cC^	16.7 ± 0.17 ^cD^	17.5 ± 0.2 ^bE^	18.1 ± 0.17 ^bF^	18.5 ± 0.26 ^bG^	19.6 ± 0.2 ^b^	-	-	-	-
	1% NE-MAP1	15.4 ± 0.26 ^aA^	15.3 ± 0.25 ^abcA^	15.2 ± 0.17 ^aA^	15.4 ± 0.35 ^abcA^	15.6 ± 0.26 ^aAB^	15.5 ± 0.35 ^abA^	15.6 ± 0.1 ^aAB^	15.3 ± 0.25 ^aA^	16 ± 0.15 ^aB^	16.8 ± 0.1 ^aC^	16.9 ± 0.20 ^aCD^	17.3 ± 0.40 ^aDE^	17.5 ± 0.32 ^aE^	18.3 ± 0.30 ^aF^	18.4 ± 0.41 ^aF^	19.1 ± 0.15 ^aG^	20 ± 0.35 ^aH^
	1% NE-MAP2	15.5 ± 0.26 ^aAB^	15 ± 0.20 ^aA^	15.4 ± 0.05 ^aAB^	15 ± 0.30 ^aA^	15.6 ± 0.43 ^aAB^	15.5 ± 0.20 ^abAB^	15.6 ± 0.2 ^aB^	15.3 ± 0.32 ^abAB^	16.4 ± 0.20 ^bcC^	16.7 ± 0.1 ^aC^	16.8 ± 0.51 ^aC^	17.4 ± 0.52 ^aD^	17.6 ± 0.30 ^aD^	18.3 ± 0.1 ^aE^	18.5 ± 0.17 ^aE^	19.3 ± 0.11 ^aF^	20.1 ± 0.30 ^aG^
	1% NE-MAP3	15.3 ± 0.32 ^aAB^	15 ± 0.25 ^abA^	15.4 ± 0.15 ^aAB^	15.3 ± 0.1 ^abAB^	15.3 ± 0.1 ^aAB^	15.4 ± 0.32 ^aAB^	15.4 ± 0.20 ^aAB^	15.7 ± 0.20 ^bBC^	16.2 ± 0.20 ^abCD^	16.7 ± 0.20 ^aDE^	16.8 ± 0.40 ^aE^	17 ± 0.37 ^aEF^	17.5 ± 0.1 ^aF^	18.1 ± 0.66 ^aG^	18.5 ± 0.43 ^aG^	19.4 ± 0.26 ^aH^	20 ± 0.41 ^aI^

MAP1—80% O_2_ + 20% CO_2_; MAP2—70% O_2_ + 30% CO_2_; MAP3—60% O_2_ + 20% CO_2_ + 20% N_2_. Values are presented as mean ± standard deviation. Within each row, means with the same uppercase letter are not significantly different (*p* > 0.05). Within each column, means with the same lowercase letter are not significantly different (*p* > 0.05).

## Data Availability

The original contributions presented in the study are included in the article/Supplementary Materials, further inquiries can be directed to the corresponding authors.

## Supplementary Materials

The following supporting information can be downloaded at: https://www.mdpi.com/article/10.3390/foods13213504/s1, Table S1: The natural antimicrobial compounds and their composition.
