# Supplementary material for: UV-C-Activated Riboflavin Crosslinked Gelatin Film with Bioactive Nanoemulsion for Enhanced Preservation of Fresh Beef in Modified Atmosphere Packaging
Source: Foods. 2024 Oct 31;13(21):3504. doi: 10.3390/foods13213504 (PMC11544885; doi:10.3390/foods13213504)
Supplement: Supplementary file 1 [file foods-13-03504-s001.zip › foods-3272712-supplementary.pdf]

**Table S1:** The natural antimicrobial compounds and their composition.

| Common name | Chemical composition |
|---|---|
| Mediterranean EO* | Thymol (14.55 %), carvacrol (39.45 %), γ-terpinene (11.2 %), α-terpinene (1.6 %), myrcene (1.45 %) and p-cymene (21.05 %) |
| German thyme EO | Thymol (50.35 %), carvacrol (3.39 %), Linalool (3.89 %), β-caryophyllene (1.30 %), Myrcene (1.82 %), α-terpinene (1.53 %), γ-terpinene (10.02 %) and p-cymene (18.43 %) |
| Vietnamese cinnamon EO | Trans-cinnamic aldehyde (90.08 %), cinnamyl acetate (4.6 %) and d-methoxy cinnamate aldehyde (7.89 %) |
| Citrus extract | Polyphenols (3.36 %) and flavonoids (0.62 %) |
